# Editorial: Dementia in Primary Care

**DOI:** 10.3389/fmed.2022.963857

**Published:** 2022-06-24

**Authors:** Ferdinando Petrazzuoli, Hein van Hout, Marieke Perry

**Affiliations:** ^1^Department of Clinical Sciences, Center for Primary Health Care Research, Lund University, Lund, Sweden; ^2^Department General Practice & Medicine for Older Persons Vrije Universiteit, Amsterdam University Medical Center, Amsterdam, Netherlands; ^3^Radboud University Nijmegen Medical Centre, Nijmegen, Netherlands

**Keywords:** dementia, family medicine, general practice, Research Topic, caregiver burden

## Introduction

By the year 2050, ~131 million will be affected by dementia and Primary Care Physicians (PCPs) will certainly play a crucial role in identifying and managing this severe condition (1).

Dementia is a clinical syndrome caused by a variety of mainly neurodegenerative diseases, including symptoms on cognitive, emotional/behavioral (depression, anxiety, agitation) and physical domains, that impact daily functioning (2). Mild Cognitive Impairment (MCI) represents similar but less severe symptoms that do not (yet) interfere with activities of daily functioning. The introduction of the terms major and minor neurocognitive disorders attempts to help reduce the stigma associated with the word dementia (3).

Cognitive impairment due to dementia or MCI in a community setting is a challenge for the health-care services, the clinicians, and the families of patients.

## Population Screening and Early Symptoms of Dementia

Recently a cross-sectional study from a Southern European setting (Greece) (Bertsias et al.) provided useful information about the extent and related co-morbidities of cognitive impairment due to dementia/MCI. In a total sample of 3,140 participants, these authors found low MMSE scores in 645 (20.5%) participants. Among participants with low MMSE scores nearly half of them underwent comprehensive neuropsychiatric evaluation and of these (53.8%) were diagnosed with MCI and (34.3%) with dementia, in the remainder the cognitive problems were not confirmed. The Greek authors also identified certain chronic illness-complexes that were associated with low MMSE scores within the context of primary care consultation. New physical early signs of dementia are currently being investigated, one of these is the presence of abnormalities in postural movements assessed objectively with Virtual Reality Technology (Imaoka et al.). Can we consider postural control impairment an early sign of the preclinical stage of dementia? We wonder if this technique can be widely implemented in primary care. Should primary care diagnostic services start offering virtual reality if it appears to be effective? But, above all, do we think we need to detect preclinical dementia when we are not yet able to promptly detect most of the clinical cases?

## Dementia, Comorbidities and the Burden of Dementia

Prevalence of comorbidity in older people with dementia is high, especially cardiovascular diseases and depression. Individuals with depression or severe depressive symptoms experience faster cognitive decline over time (4). In vascular dementia, living with post-stroke conditions (Tang et al.) combined with memory impairment can have negative effects on the stroke-survivor and their family when they are discharged from hospital and return at home. Primary care physicians are aware of the huge burden involved with this condition and dementia management is often characterized as an “Unburdening process” with the goal to relieve the dementia burden; primary care doctors are aware of the low effectiveness of dementia drugs but still tend to use them, sometimes outside of guideline indications, in an ultimate attempt to help patients and their caregivers (5).

## Effectiveness of the Therapeutic Approaches

There is no effective pharmacological cure for dementia (6). The High Authority of Health in France even decided to stop reimbursement of anti-Alzheimer drugs because of lack of clinically relevant effects and the risk of side effects. Dementia management is recommended to focus on well-being including meaningful daily activities and caregiver support. People with dementia have complex problems and symptoms in many domains. Interventions should be individualized and consider the person as a whole, as well as their family carers. Evidence is accumulating for the effectiveness, at least in the short term, of psychosocial interventions tailored to the patient's needs, to manage neuropsychiatric symptoms. Evidence-based interventions for carers can reduce depressive and anxiety symptoms over years and be cost-effective.

## Perceived Risk of Developing dementia

Literature shows that an increased perceived (Hajek and König) own risk of developing dementia is associated with younger age, higher education, poor self-rated health, an increased number of chronic diseases, and an increased agreement that a diagnosis of dementia would ruin their life. An increased perceived modifiability of memory deterioration is associated with higher education, and not being employed, but not to health-related variables, therefore patients are often aware of this “Healthy Brains in Healthy Hearts” relationship.

## Role of Primary Care in the Post Diagnostic Stage

In the post-diagnostic stage PCPs can intervene effectively, using various (non-drug) treatment measures and referrals to support services (1). This should include not only the persons with dementia, but also their caregivers (7). The impact of caregiver burden includes neglected personal health, depression, anxiety, financial problems and employment losses (7, 8).

Additionally, caregivers and their close relatives are more vulnerable to social isolation and psychological distress resulting from the heavy demands of care-giving and the challenges of managing dementia, in particular the challenges of managing the behavioral and psychological symptoms of dementia.

Intermediate care which is defined as healthcare occurring between traditional primary (community) and secondary (hospital) care settings can be helpful in alleviating caregiver burden and preventing caregiver burn out. There are several types of intermediate care, the most popular are: integrated at-home services, respite/relief services, day care centers, and finally after the carers have 'given up', other options would be nursing and residential homes.

Psychoeducation is one of the most effective interventions for caregivers and recently e-education has also proved effective and is becoming available in many countries and languages (9). Case management can also positively impact carers' quality of life (10). In a qualitative study conducted in Ireland (Bourque and Foley) PCPs stated that they consider dementia care to be a complex and challenging aspect of primary care. “Service delivery must be reconfigured.” This will necessitate adequate financial resourcing and the restructuring of community-based dementia care services and facilitate interprofessional collaboration in primary care (11).

## Social Health and Other Modifiable Risk Factors

Living alone increases by 50% the risk of developing dementia while participation in physical leisure activities seems to reduce the risk of dementia in subjects with MCI (6). One of the best approaches to support people who suffer from social isolation is “Social Prescribing”, which can be defined as a mechanism for linking patients with non-clinical sources of support within the community. Engaging patients in arts, social activities and interacting within their communities makes them feel more involved, more confident, and more resilient. Relationships are a basic human need (12).

Legal issues for dementia sufferers and families are also extremely important and PCPs should check that legal issues are being dealt with by the family. Advance life decisions conversations need to become more common.

## Advance Care Planning

In our aging society it is very likely that people in their lives, have directly experienced the enormous burden of the family caregiver and are therefore aware of the problems associated with the severe stages of dementia. In a Dutch study (Azizi et al.) it was found that conversations on advance care planning in persons with dementia in general practice were rare, in particular among persons with a non-Western background and often these conversations started long after diagnosis.

In the future, these issues need to be addressed more properly and require PCP training to reduce the huge burden for patients and caregivers in the advanced stage of dementia (13).

## Author Contributions

FP made the first draft. All authors read and approved the final manuscript.

## Conflict of Interest

The authors declare that the research was conducted in the absence of any commercial or financial relationships that could be construed as a potential conflict of interest.

## Publisher's Note

All claims expressed in this article are solely those of the authors and do not necessarily represent those of their affiliated organizations, or those of the publisher, the editors and the reviewers. Any product that may be evaluated in this article, or claim that may be made by its manufacturer, is not guaranteed or endorsed by the publisher.
